# Symptom Severity and Demoralization Associated With Functional Ability Impairment in Oral Cancer Patients

**DOI:** 10.1097/jnr.0000000000000771

**Published:** 2026-09-11

**Authors:** Bing-Shen HUANG, Su-Ying YU, Hui-Ping LIN, Ying-Mai KUNG, Ya-Lan CHANG, Chien-Yu LIN, Ching-Fang CHUNG, Shu-Ching CHEN

**Affiliations:** 1 Department of Medicine, College of Medicine, Chang Gung University, Taoyuan, Taiwan; 2 Department of Radiation Oncology, and Proton and Radiation Therapy Center, Chang Gung Medical Foundation, Linkou Chang Gung Memorial Hospital, Taoyuan, Taiwan; 3 Department of Nursing, Koo Foundation Sun Yat-Sen Cancer Center, Taipei, Taiwan; 4 Department of Nursing, College of Nursing, Chang Gung University of Science and Technology, Taoyuan, Taiwan; 5 College of Nursing, Florida State University, Florida, United States; 6 Department of Nursing, Linkou Chang Gung Memorial Hospital, Taoyuan, Taiwan; 7 Department of Medical Imaging and Radiological Sciences, College of Medicine, Chang Gung University, Taoyuan, Taiwan; 8 School of Nursing and Center for Health and Geriatric Care Research and Development, College of Nursing, Chang Gung University of Science and Technology, Taoyuan, Taiwan; 9 School of Nursing, College of Medicine, Chang Gung University, Taoyuan, Taiwan

**Keywords:** cancer, head and neck cancer, oral cancer, recurrence, symptom, demoralization, functional impairment

## Abstract

**Background:**

Oral cancer (OC) is the most common head and neck cancer and is associated with the highest recurrence rate of cancers of this type. OC recurrence and its treatment may cause symptoms and demoralization that result in impaired functional ability.

**Purpose:**

This study was designed to: (1) assess symptom severity, demoralization, and functional impairment in patients with recurrent OC, (2) identify factors associated with functional impairment, and (3) compare functional impairment among patients with recurrent OC with respect to work status and number of cancer recurrences.

**Methods:**

In this cross-sectional study, data collected by convenience sampling of patients with recurrent OC at a cancer center in Northern Taiwan were used. Assessments included the World Health Organization Disability Assessment Schedule 2.0, the Symptom Severity Scale, the Demoralization Scale, the Karnofsky Performance Status Index, and demographic and clinical information.

**Results:**

Being of older age, working as a laborer, having a higher level of symptom severity, being affected by demoralization, or having a higher level of demoralization were identified as significant risk factors for functional impairment. Unemployed participants faced a higher risk of functional impairment in the realms of cognition, mobility, self-care, getting along with people, life activities, and participating in society than their employed peers.

**Conclusions:**

The findings indicate that patients with recurrent OC who are older, do manual labor, have more severe symptoms, or have higher levels of demoralization face a significantly higher risk of functional impairment. Health care professionals should be aware of patients’ age, symptoms, and demoralization status and monitor related changes during disease-free survival.

## Introduction

Oral cancer (OC), defined as malignant neoplasia of the oral cavity, is the most common of the head and neck cancers (Colevas et al., 2025). Globally, OC has the thirteenth-highest incidence of all cancers, with about 377,713 people diagnosed each year. Betel quid chewing in combination with smoking and drinking has been observed to be prevalent among patients with OC in South Asia, East Africa, and the Pacific region, including Taiwan (Centers for Disease Control and Prevention, n.d.). In Taiwan, OC is the fourth most common cancer among males, with ∼4,100 new cases annually (Taiwan Cancer Registry, 2022). Surgery, chemotherapy, radiation therapy (RT), and concurrent chemoradiation therapy (CCRT) are standard treatments (National Comprehensive Cancer Network (NCCN), 2025). The five-year survival rates for OC stages I, II, III, and IV are 85%, 70%, 55%, and 35%, respectively (Cancer Research UK, n.d.). OC treatment often has adverse acute and late effects (Taiwan Cancer Registry, 2025), which may cause symptoms, demoralization, and varying degrees of functional impairment (T. G. Chang et al., 2022; Peng et al., 2024).

A substantial proportion of patients with OC experience significant symptom distress. The most prevalent symptoms among oral cancer patients postoperatively include difficulty swallowing/chewing, difficulty with voice/speech, and mucostitis (Ou et al., 2022). Patients who receive RT or CCRT report tiredness, poor well-being, and lack of appetite, with the most severe symptoms occurring during the treatment phase (Allen-Ayodabo et al., 2019).

Demoralization, a clinical state characterized by hopelessness, helplessness, and a loss of meaning in life, is common among patients with cancer and among those with recurrent or metastatic cancer (Grassi & Nanni, 2016; Tang et al., 2025). T. G. Chang et al. (2022) reported the mean demoralization score among inpatients with oral cancer to be 27.2 (*SD* = 16.8), with 35.5% categorized as having a high level of demoralization (Demoralization Scale [DS] >30). High demoralization has been associated with being older, female, or married and having depression (Lin & Her, 2024), a lower income, lower education, higher symptom burden (Tang et al., 2025), poor quality of life, low satisfaction with spiritual needs, or high risk of suicidal ideation (Y. L. Chang et al., 2022).

Functional impairment is defined as difficulty performing the activities of daily living necessary to maintain health and meet basic needs (Leidy, 1994). Y. H. Lee et al. (2020) found that patients with head and neck cancer (HNC) with impaired swallowing had lower WHODAS 2.0 overall and domain scores, especially in the domain of participating in society. Y. H. Lee et al. (2019) found that HNC survivors who were unemployed reported a mean WHODAS 2.0 score of 46.27 (range, 0–100), with decreased functional ability in getting along with people, participating in society, life activities, getting around, performing self-care, and understanding and communicating. H. H. Lee et al. (2017) found that colorectal cancer survivors reported a mean WHODAS 2.0 score of 46.0 (range, 0–100), with decreased levels of participating in society, getting around, performing household activities, performing self-care, getting along with people, and understanding and communicating. Patients more likely to report functional impairment were those affected by demoralization (Hong et al., 2022), comorbidities (Cheung et al., 2015), and physical–mental comorbidities (Ferro et al., 2023).

Although this topic has been investigated in previous studies, most of the cohorts have been from Korea (H. H. Lee et al., 2017; Y. H. Lee et al., 2019; Y. H. Lee et al., 2020) and included patients with head and neck cancer or colorectal cancer. Cultural and social differences among countries may lead to variations in symptom severity, demoralization, and functional impairment among patients with recurrent OC. These issues have been inadequately investigated in patients with recurrent OC in Taiwan. Based on the results of a literature review, the hypotheses of this study were that: (1) patients with recurrent OC experience greater symptom severity, higher levels of demoralization, and reduced functional ability, and (2) patients who are unemployed or have a higher cancer recurrence frequency face a higher risk of functional impairment. As work status and the number of cancer recurrences may be associated with different levels of functional impairment, the aims of this study were to (1) assess symptom severity, demoralization, and functional impairment in patients with recurrent OC, (2) identify factors associated with functional impairment, and (3) compare functional impairment among patients with recurrent OC with respect to work status and number of cancer recurrences.

## Methods

### Design and Sample

For this cross-sectional study, the participants were recruited using convenience sampling from the outpatient radiation department of Chang Gung Memorial Hospital in northern Taiwan between August 2021 and July 2024. Inclusion criteria were: (1) 18 years or older, (2) a diagnosis of primary, recurrent, or metastatic OC, (3) regular follow-up in the outpatient department through physician visits, and (4) for human–subject protection, a Karnofsky Performance Status (KPS) score (Clancey, 1995) of ≥60. Exclusion criteria were: (1) any unstable systemic disease (other underlying disease) and (2) any condition likely to cause discomfort during the research interview.

### Ethical Considerations

Approval was granted by the Ethics Committee of Chang Gung Medical Foundation, under approval code number 202002176B0. The participants were informed of the study purpose, procedure, data collection, potential risks, and right to refuse participation or withdraw from the study. Informed written consent was obtained from participants upon enrollment. The participants agreed to fill out a set of questionnaires designed to assess symptom severity, demoralization, and functional impairment. Permission for data collection included signing an informed consent document. All consent forms and questionnaires were preserved for five years in a dedicated and secure locked cabinet in the principal investigator’s office. Patients who met the inclusion criteria were advised of their right to refuse study participation and withdraw from the study at any time with no effect on their treatment.

### Data Collection

Potentially eligible patients were initially identified using patient registration lists at the outpatient radiation departments of the participating hospitals. All eligible patients were recruited to participate in this study by invitation from the principal investigator, co-principal investigators, or research nurse. The study data were collected using a set of questionnaires administered face-to-face by a research nurse in a private setting; the process took about 10–15 minutes.

### Measures

#### World Health Organization Disability Assessment Schedule 2.0

Functional impairment was measured in this study using the World Health Organization Disability Assessment Schedule 2.0 (WHODAS 2.0). The original English version of the WHODAS 2.0 developed by Üstün, Chatterji, et al. (2010) consists of 12 items designed to measure six domains. These include: cognition (two items), covering functions such as concentrating, remembering, problem solving, learning and communicating; mobility (two items), covering functions such as standing, moving around inside the home, getting out of the home, and walking a long distance; self-care (two items), covering patient hygiene, dressing, eating, and staying alone; getting along (two items), covering interaction with a spouse or partner, family members or close friends, and strangers; life activities (two items), covering household, work, and school activities; and participation (two items), covering participation in community activities and society (Üstün, Kostanjsek, et al., 2010). The summed total score is converted into a standardized score ranging from 0 to 100, with higher scores reflecting higher functional impairment (Üstün, Kostanjsek, et al., 2010). A cutoff point of <27.81 was identified as a predictor of head and neck cancer patient return to work during survival (Y. H. Lee et al., 2019). Functional impairment was assessed using the Chinese version of the WHODAS 2.0, for which the Cronbach’s α and ICC were .73–.99 and .8–.89, respectively (T. Y. Chiu et al., 2014), which satisfied content validity, concurrent validity, and construct validity requirements. In this study, Cronbach’s α was .85.

#### Demoralization Scale

Demoralization was measured in this study using the Demoralization Scale (DS). The original English version of the DS was developed by Kissane et al. (2004). This 24-item questionnaire includes patient responses to questions covering the following 5 domains: loss of meaning (five items), dysphoria (five items), hopelessness (six items), helplessness (four items), and sense of failure (four items). All items are scored using a 5-point Likert Scale, with scores ranging from 0 (*never*) to 4 (*all the time*) and higher total scores indicating a higher level of demoralization (Kissane et al., 2004). The total possible score ranges from 0 to 96, and scores of 30 or more indicate a high level of demoralization (Kissane et al., 2004). The Chinese DS has shown satisfactory psychometric characteristics in a previous cancer-related study (Cheng et al., 2019), and the DS has been regularly used to evaluate demoralization in patients with cancer (Wang et al., 2023). The Cronbach’s α for the DS in this study was .94.

#### Symptom Severity Scale

Symptom severity was measured in this study using the Symptom Severity Scale (SSS). The 13-item SSS is designed to assess HNC-specific symptoms. Each item is scored on an 11-point scale ranging from 0 to 10, with 10 representing “*extremely severe symptom*” and 0 representing “*no such symptom at all*” (Hu et al., 2019). In this study, the Cronbach’s α value for the SSS was .90.

#### Karnofsky Performance Status Index

Physical performance was measured using the Karnofsky Performance Status (KPS). The original English version of the KPS Index developed by Karnofsky (Clancey, 1995) is commonly used to assess physical performance in cancer patients (Chow et al., 2020). The KPS instrument is a single-item measure with a score ranging from 0% (dead) to 100% (normal physical performance; Clancey, 1995).

#### Demographic and clinical characteristics

The demographic variables collected for this study included: age, sex, occupation status, marital status, educational level, religion, and family annual income (in New Taiwan dollars, NT$). The clinical variables assessed included: body mass index (BMI), tumor site, cancer stage, and medical treatment.

### Data Analysis

All of the data were tested for skewness before conducting the statistical analyses (inferential statistics and parametric testing). Continuous data are reported in terms of mean values and *SD*, while categorical data are presented as percentages and frequencies. To compare normative data, continuous data are presented as mean values and *SD*. Pearson’s product-moment correlation was used to analyze correlations between functional disability and selected variables (old age [no vs. yes], body mass index [≥25 vs. <25 kg/m^2^], manual labor work [no vs. yes], Symptom Severity Scale [SSS], and Demoralization Scale [DS]). Logistic regression analysis was used to identify factors associated with functional impairment (dependent variable). Functional impairment was categorized as normal to mild (<27.81) or moderate to severe (>27.82; Y. H. Lee et al., 2019). The independent variables included old age (no vs. yes), body mass index (≥25 vs. <25 kg/m^2^), manual labor work (no vs. yes), symptom severity (mean SSS score), and demoralization (sum of the DS score). The independent-samples *t* test was used to compare functional impairment and the six specific domains of functional impairment based on work status (unemployed vs. employed) and number of cancer recurrences (1 vs. ≥2).

## Results

### Patient Characteristics

Of the 298 eligible patients with OC approached, 10 refused to participate due to no interest or no time, with 288 participating in this study (response rate = 96.6%). The demographic and clinical characteristics of the patient cohort at baseline are shown in Table 1.

**Table 1:** Demographic and Clinical Characteristics at Time of Recurrence (*N*=288)

Variable	*n* (%)
Age (years, *M* and *SD*)	57.32 (7.16)
Sex	
Male	283 (98.3)
Female	5 (1.7)
Work status	
Unemployed	190 (66.0)
Employed	98 (34.0)
Marital status	
Living with a partner	244 (84.7)
Living alone	44 (15.3)
Educational level	
Elementary	46 (16.0)
Junior high	96 (33.3)
Senior high	116 (40.3)
College and above	30 (10.4)
Religion	
None	7 (2.4)
Buddhism/Taoism	279 (96.9)
Christianity/Catholicism	2 (0.7)
Type of occupation	
Unskilled/ semiskilled worker	72 (25.0)
Skilled worker	106 (36.8)
Clerk, shop owner, farm owner	103 (35.8)
Semiprofessional	5 (1.7)
Professional	2 (0.7)
Family annual income (NT; USD)	
≤300,000 (USD 9,810.33)	36 (12.5)
300,001–600,000 (USD 9,810.37–19,620.67)	113 (39.2)
600,001–1,000,000 (USD19,620.70–32,701.11)	108 (37.5)
1,000,001–1,500,000 (USD 32,701.14–49,051.67)	23 (8.0)
>1,500,000 (USD 49,051.67)	8 (2.8)
No. cancer recurrences	
1	217 (75.3)
≥2	71 (24.7)
Time since most recent recurrence (years)	
<1	85 (29.5)
1–3	121 (42.0)
3.1–5	38 (13.2)
>5	44 (15.3)
Body mass index	
Severely underweight	2 (0.7)
Underweight	12 (4.2)
Normal	189 (65.6)
Overweight	69 (24.0)
Obese	16 (5.6)
Tumor site of initial diagnosis	
Lip	4 (1.4)
Buccal mucosa	53 (18.4)
Oral tongue	87 (30.2)
Gingivae	97 (33.7)
Mouth floor	4 (1.4)
Hard plate	32 (11.1)
Retromolar	11 (3.8)
Cancer stage at initial diagnosis	
I	25 (8.7)
II	48 (16.7)
III	35 (12.2)
IV	180 (62.5)
Medical treatment of initial diagnosis	
Surgery + RT	66 (22.9)
Surgery + CT	5 (1.7)
Surgery + CCRT	149 (51.7)
RT	35 (12.2)
CCRT	33 (11.5)
KPS score (level, *M* and *SD*)	88.09 (4.20)
81–90	236 (81.9)
71–80	49 (17.0)
60–70	3 (1.0)

*Note.* RT = radiotherapy; CT = chemotherapy; CCRT = combined chemotherapy and radiotherapy; KPS = Karnofsky performance score.

### Levels of Symptom Severity, Demoralization, and Functional Impairment

The mean symptom severity (SSS score) was 6.19 (*SD*=1.72). The mean demoralization (DS score) was 22.47 (*SD* = 11.17). Mean subscale scores were: loss of meaning, 3.67(*SD* = 2.73); dysphoria, 5.20 (*SD* = 2.77); hopelessness, 5.86 (*SD* = 3.77); helplessness, 3.56 (*SD* = 1.74); and sense of failure, 4.18 (*SD* = 2.21). The mean functional impairment (WHODAS 2.0 score) was 30.22 (*SD* = 9.10). Mean subscale scores were: cognition, 30.03 (*SD* = 11.29); mobility, 30.64 (*SD* = 13.23); self-care, 25.35 (*SD* = 2.53); getting along with people, 31.94 (*SD* = 13.43); life activities, 29.90 (*SD* = 11.01); and participation in society, 32.42 (*SD* = 17.33; Table 2).

**Table 2:** Scores for Symptom Severity, Demoralization, and Functional Impairment (N=288)

Variable	Mean (*SD*)	*n* (%)	Range	Theoretical Score Range
Symptom Severity Scale	6.19 (1.72)		0–10	2.50–9.13
Demoralization Scale	22.47 (11.17)		0–60	0–96
Loss of meaning	3.67 (2.73)		0–12	0–20
Dysphoria	5.20 (2.77)		0–14	0–20
Hopelessness	5.86 (3.77)		0–20	0–24
Helplessness	3.56 (1.74)		0–10	0–16
Sense of failure	4.18 (2.21)		0–14	0–16
High level of demoralization				
No (<30)		231 (80.2)		
Yes (≥30)		57 (19.8)		
Functional impairment (WHODAS 2.0)				
No (≤27.81)		161 (55.9)		
Yes (≥27.82)		127 (44.1)		
Functional impairment (WHODAS 2.0)	30.22 (9.10)		23–67	0–100
Cognition	30.03 (11.29)		25–75	0–100
Mobility	30.64 (13.23)		25–100	0–100
Self-care	25.35 (2.53)		25–50	0–100
Getting along with people	31.94 (13.43)		25–100	0–100
Life activities	29.90 (11.01)		25–75	0–100
Participant in society	32.42 (17.33)		13–88	0–100

*Note.* WHODAS 2.0 = World Health Organization Disability Assessment Schedule 2.0.

### Factors Associated With Functional Impairment

One hundred twenty-seven participants (44.1%) reported functional impairment. Functional impairment (normal to mild vs. moderate to severe) was used as the dependent variable in the logistic regression analysis, and the results showed those who were > 65 years of age (β = 2.093, *p* < .01), worked as laborers (β = 0.674, *p* < .01), had a higher level of symptom severity (β = 0.212, *p* < .05), or had a higher level of demoralization (β = 0.162, *p* < .01) to be more likely to be affected by functional impairment (Table 3).

**Table 3:** Logistic Regression Analysis of Factors Related to Functional Impairment (*N*=288)

Variable	Beta	*SE*	Wald test	*p*	Odds Ratio/95% CI
Old age (no vs. yes)	2.093	0.516	16.446	.001	0.123 [0.045, 0.339]
Body mass index (≥25 vs. <25 kg/m^2^)	0.137	0.354	0.150	.001	1.147 [0.574, 2.294]
Manual labor work (no vs. yes)	0.674	0.210	10.271	.001	0.510 [0.337, 0.770]
Symptom severity	0.212	0.090	5.545	.001	1.236 [1.036, 1.475]
Demoralization	0.162	0.023	51.592	.001	1.176 [1.125, 1.229]
Constant	3.570	0.889	16.143	.001	0.028 [—]

*Note.* χ^2^ = 132.771, *p*<.001, Nagelkerke *R* ^*2*^ = .495.

Input independent variable: covariates included old age (no vs. yes), body mass index (≥25 vs. <25 kg/m^2^), manual labor work (no vs. yes), symptom severity (continuous score, using the mean score of the Symptom Severity Scale), and demoralization (continuous score, using the sum score of the Demoralization Scale).

### Functional Impairment According to Work Status and Number of Cancer Recurrences

Of the 288 participants, 190 were unemployed and 98 were employed. The independent-samples *t* test results show those who were unemployed had significantly higher scores for functional impairment (*t* = −5.424; *p* < .01), cognition (*t* = −4.074; *p* < .01), mobility (*t* = −4.265; *p* < .01), self-care (*t* = −1.775; *p* < .01), getting along with people (*t* = −4.113; *p* < .01), life activities (*t* = −4.139; *p* < .01), and participation in society (*t* = −4.348; *p* < .01) than their employed peers.

Also, 217 of the participants had experienced one cancer recurrence, while the remainder (71) had experienced two or more. The independent-samples *t* test results show those who had experienced two or more cancer recurrences earned higher scores for functional impairment, mobility, self-care, getting along with people, life activities, and participation in society, although the between-group differences in scores for these variables did not reach statistical significance (Table 4).

**Table 4:** Differences in Functional Impairment by Work Status and Number of Cancer Recurrences (*N*=288)

	Work Status				No. Cancer Recurrences			
	Unemployed (*n*=190)	Employed (*n*=98)			1 (*n*=217)	≥2 (n=71)		
Variable	Mean (*SD*)	Mean (*SD*)	*t*	*p*	Mean (*SD*)	Mean (*SD*)	*t*	*p*
Functional impairment (WHODAS 2.0)	32.15 (10.33)	26.47 (3.99)	6.679	.001	30.14 (8.94)	30.36 (9.49)	−0.192	.848
Cognition	32.96 (12.73)	27.30 (6.31)	5.050	.001	31.06 (11.11)	30.98 (11.70)	0.051	.959
Mobility	32.96 (15.48)	26.15 (4.43)	5.635	.001	30.41 (11.86)	31.12 (15.74)	−0.423	.672
Self-care	25.53 (3.11)	25.00 (0.00)	2.336	.001	25.26 (2.53)	25.53 (2.54)	−0.861	.390
Getting along with people	34.34 (15.48)	27.30 (5.77)	5.568	.001	32.15 (13.98)	31.52 (12.26)	0.376	.707
Life activities	31.78 (12.77)	26.28 (4.57)	5.315	.001	29.77 (10.25)	30.19 (12.49)	−0.302	.763
Participant in society	35.33 (17.80)	26.79 (14.91)	4.306	.001	32.22 (16.23)	32.85 (19.49)	−0.289	.773

*Note.* WHODAS 2.0 = World Health Organization Disability Assessment Schedule 2.0; higher scores reflect greater functional impairment.

## Discussion

The findings of this study show that the participants with recurrent OC experienced the greatest functional impairments with respect to participation in society, interpersonal relationships, mobility, cognition, life activities, and self-care. These results support those of previous studies (H. H. Lee et al., 2017; Y. H. Lee et al., 2020) that reported functional limitations as most prominent in the domains of social participation and interpersonal interaction. The observed impairments in social participation and getting along with others may be attributed to both physical and psychosocial challenges. Disfigurement, speech difficulties, and eating problems after cancer treatment may lead to social withdrawal, loss of confidence, and stigma, all of which are known to affect social engagement. Health care professionals should routinely assess the functional status of their patients, focusing on social participation and interpersonal functioning. The early implementation of rehabilitation programs, including speech therapy, psychosocial counseling, physical activity training, and peer support groups, may help patients regain functional independence and improve social reintegration.

In this study, the most common categories of demoralization reported by patients with recurrent OC were hopelessness, dysphoria, and sense of failure. These results are similar to those of Kang et al. (2023), who found sense of failure, hopelessness, and dysphoria associated with the worst levels of demoralization. However, in this study, 19.8% of patients with recurrent OC perceived their level of demoralization as high, which is much fewer than the 49% reported by Ghiggia et al. (2021). These between-study differences in level of demoralization may be attributable to differences in disease phase and hospitalization setting. In this study, patients with recurrent or metastatic OC and those with recurrent OC were investigated in their disease-free survival phase. Also, whereas Ghiggia et al. (2021) studied hospitalized end-of-life patients with a variety of cancer types, the patients with recurrent OC in this study were attendees of outpatient departments. Clinical care requires awareness of the stages of the cancer journey and the varying levels of demoralization associated with these stages.

The results of this study show that the participants over 65 years of age were more likely to experience impaired functional ability. Nearly one-fifth of our participants were older than 65 years, and many had experienced cancer recurrence within 1–3 years before the study. These findings suggest older patients may benefit from a survivorship care approach that includes active professional surveillance, early intervention, and tailored guidance to strengthen physical function. Such an approach may help these vulnerable individuals cope with treatment-related adverse effects, cancer recurrence, ageing, frailty, and the resulting decline in physical function.

In addition, the findings of this study show that those participants with more severe symptoms exhibited greater impairments in functional ability. These findings are consistent with previous studies reporting that cancer patients with more severe symptoms tend to experience higher levels of functional limitation (Othman et al., 2023; Yang et al., 2022). These results highlight the importance of routinely assessing patient functional status and implementing early interventions such as prehabilitation, physical activity programs, and dietary support to maintain optimal nutritional and functional status before and during treatment.

Also, the results of this study show that participants who did not work doing manual labor were more likely to have greater functional impairment than those who did, and that participants who were unemployed earned significantly higher scores for functional impairment and on each domain of functional impairment than those who were employed. These results are consistent with the findings of several previous studies (Chen et al., 2025; S. E. Chiu et al., 2023) reporting that most patients had difficulty in social participation and getting along with others, posing barriers to returning to work after treatment. Therefore, health care professionals should assess patients' physical function and provide physical activity guidance to improve their physical ability during treatment for recurrence.

Finally, the participants who had experienced two or more cancer recurrences earned higher scores for functional impairment, mobility, self-care, getting along with people, life activities, and participation in society than their peers who had experienced one cancer recurrence. However, the between-group differences in these scores did not reach statistical significance, which does not support the related hypothesis (Y. H. Lee et al., 2019). Half of the participants had undergone surgery combined with CCRT, one-third had two or more cancer recurrences, and the mean time since the most recent recurrence was 1–3 years. These findings suggest that late-delayed adverse effects continue to impair functional ability for 1–3 years after the initial RT (Oba et al., 2021). This finding suggests physical activity education, posttreatment surveillance, and employee counseling should be included in nursing care for patients with OC.

### Limitations and Strengths

This study was affected by several limitations. First, while baseline physical ability before treatment may have influenced the factors associated with physical impairment, this variable was not included in our analysis. This variable should be considered in future research, and its correlation with physical ability outcomes should be examined. Second, the cross-sectional design used may limit the interpretability of results. Long-term follow-up or longitudinal studies are needed to identify changes in functional impairment from the time of treatment completion until cancer recurrence. Third, the convenience sampling approach used to recruit participants may mean the results do not reflect the actual frequency of cancer recurrence. Random sampling should be used in future studies, and the effects of cancer recurrence on deformity and dysfunction should be investigated. Finally, as questionnaires were used to assess variables, the results may not accurately reflect the experiences of patients with recurrent OC. Interview guidelines should be developed and used in future studies, and qualitative and quantitative data should be considered together to add greater meaning to the responses of patients with recurrent OC.

### Conclusions

In this study, 44.1% of the participants (patients with recurrent OC) reported functional impairment. Being over 65 years of age, working as a laborer, having more severe symptoms, and having a higher level of demoralization were all associated with a higher risk of functional impairment. Moreover, being unemployed (vs. employed) was found to be associated with greater functional impairment in the categories of cognition, mobility, self-care, getting along with people, life activities, and participating in society. In light of these findings, health care professionals should monitor changes in levels of symptom severity and demoralization particularly closely in patients with OC who are older or work as laborers during disease-free survival. Also, clinical cancer care should include patient education on symptom surveillance and symptom management, guidance for physical activity, and expanded use of health care (physical therapy, nutrition consulting, social worker services, and home care) to delay physical ability decline.
